# Why Is It Thus?

**Published:** 1897-08

**Authors:** 


### WHY IS IT THUS?

In another portion of this issue will be found a careful, painstaking review of the work in Massachusetts, to which we have repeatedly called attention and scented danger ahead to the welfare of our professional standing and occupation. We strongly urge all our readers to carefully peruse this presentation of the facts by one of our staff contributors, and learn from it the lessons of danger it teaches. A better understanding by all will make more forcible in one's mind the necessity of our getting together on all movements of public import and of being at all times able to present a single plan of action for effective work, except in mere details of enforcement.

